# TikTok and Instagram as Putative Social Media in Promoting Healthy Eating Habits in Youths At-Risk for Eating/Feeding Disorders and Body Image Dissatisfaction

**DOI:** 10.3390/brainsci16040379

**Published:** 2026-03-30

**Authors:** Laura Orsolini, Giulio Longo, Teresa Cantarini, Salvatore Reina, Umberto Volpe

**Affiliations:** 1 Unit of Clinical Psychiatry, Department of Clinical Neurosciences/DIMSC, Polytechnic University of Marche, 60020 Ancona, Italy; l.orsolini@staff.univpm.it (L.O.);; 2 Degree Course in Dietetics, Faculty of Medicine and Surgery, Polytechnic University of Marche, 60020 Ancona, Italy

**Keywords:** feeding and eating disorders (FEDs), social media, TikTok, Instagram, digital eating habits

## Abstract

**Highlights:**

**What are the main findings?**

- TikTok use was significantly associated with higher risk of eating and feeding disorders (FEDs) and body image concerns, and it was more frequently used by participants screening positive for FED risk.
- Both TikTok and Instagram were linked to body dissatisfaction, while overall FED risk was predicted by TikTok use, higher problematic social media use scores, exposure to body positivity/neutrality content, and watching Mukbang videos.

**What are the implications of the main findings?**

- TikTok and Instagram may represent effective communication channels for delivering prevention and educational interventions targeting young people at risk of eating disorders.
- Prevention strategies should be tailored according to specific vulnerabilities within the eating disorder spectrum, and further longitudinal research is needed to confirm effectiveness.

**Abstract:**

**Background**: The widespread use of Social Networks (SNS), particularly among youths, could promote Feeding and Eating Disorders (FEDs), but could also be a tool for implementing FED prevention strategies. This study aimed to identify which SNS could be most effective for implementing primary and secondary FED prevention. **Methodology**: A cross-sectional study was conducted via an Italian population-based survey, distributed using a snowball sampling strategy. The survey included 283 participants aged 18–35 by using the Bergen Social Media Addiction Scale (BSMAS), the SCOFF screening tool for FEDs, items from the Body Uneasiness Test (BUT), and the Mukbang Addiction Scale (MAS). **Results**: The sample was predominantly female (69.3%). Participants screening positive on the SCOFF were more frequently TikTok users. Stepwise logistic regression analysis showed that TikTok use was associated with SCOFF positivity (OR = 1.9) and body image concerns (e.g., spending a lot of time in front of the mirror; OR = 1.9). Instagram use was associated with body image dissatisfaction (OR = 3.9). In the overall sample, the likelihood of screening positive on the SCOFF was associated with TikTok use (OR = 1.7), higher BSMAS scores (OR = 1.1), exposure to body positivity/neutrality content (OR = 1.9), and watching Mukbang videos (OR = 1.8). **Conclusions**: TikTok and, to a lesser extent, Instagram appear to be widely used by young individuals vulnerable to FEDs and body image dissatisfaction. These platforms may therefore represent strategic channels for delivering educational and preventive interventions targeting eating behaviors and body image among young people. Further longitudinal research is needed to clarify causal relationships and evaluate the effectiveness of SNS-based prevention strategies.

## 1. Introduction

FEDs (Feeding and Eating Disorders) represent a clinical and social challenge of considerable importance, profoundly affecting the quality of life of both those who suffer from them and their entire social and family network [1]. Similarly, the widespread use of Social Networking Sites (SNS), especially among younger populations, has potentially facilitated the creation and consultation of accounts dedicated to nutrition and diet [2]. These SNS accounts are not always managed by individuals who are adequately trained and informed about proper dietary and nutritional lifestyles. Therefore, these accounts instead promote and encourage unhealthy eating and/or nutritional habits and are ineffective in providing information and prevention on the topic of FEDs [2].

On the other hand, SNS could be a potential tool for the prevention and treatment of FEDs, especially because adolescence is a critical risk period for their onset. Nowadays, social networking is the most advanced form of online communication, and the number of users is constantly growing; in particular, according to a 2022 survey conducted by the Italian National Institute of Health (ISS), being “always online” is considered normal behavior [3]. The responsible use of SNS has a positive impact on well-being, increasing the perception of social support [4,5,6]. Nevertheless, problematic use of SNS has been associated with anxiety, depression, physical symptoms, and poorer sleep and eating habits [7]. According to the ISS survey, problematic SNS use was higher among girls in every age group (11, 13, and 15 years old), with an increase compared to data collected in 2017/2018 [3]. A Spanish study investigated the possible impact of the COVID-19 pandemic on SNS use, analyzing its association with the onset and/or maintenance of body image disorders and self-esteem in a sample of young Spanish women [8]. The study observed an increase in the amount of time spent using SNS, with an increase in Instagram use. In particular, Instagram use was associated with greater body dissatisfaction, a greater desire to be thin, and lower self-esteem, especially among youngsters [8]. Another study found that patients who tested positive for a screening tool for eating disorders (SCOFF: Sick, Control, One stone, Fat, Food) spent many more hours on SNS than negative patients, more frequently reporting that they spent this time comparing their bodies with those of the users they followed [9]. These results suggest a possible association between SNS use, body image disorders, and an increased risk of developing a drive for thinness and/or body dissatisfaction. At the same time, it emphasizes how body dissatisfaction or the drive to be thin could increase the individual’s propensity to use social networks to compare their body image with other bodies seen on the web [9]. Furthermore, other evidence suggests the possible association between the increased risk of engaging in unhealthy eating behavior and the amount of time spent on SNS, particularly when checking/searching for content related to food and/or eating behavior [10].

Overall, female adolescents seem to be the most frequent visitors of pro-eating disorder websites (pro-ana—pro-anorexia; pro-mia—pro-bulimia) [11]. These pro-ana and pro-mia sites encourage negative eating behavior, promoting a pro-anorexic approach through weight loss tips (such as laxatives, excessive exercise, calorie restriction, and diet pills), displaying ideal images of extreme thinness. Many of these websites are moderated by girls and in 58% of cases contain images encouraging weight loss [11]. Conversely, many SNS platforms, such as Instagram and TikTok, nowadays include pages promoting a positive attitude towards the body, the so-called “Body Positivity Movement”. In addition, the “Body Neutrality Movement” encourages a neutral relationship with the body, focusing on appreciating it for its functionality rather than labeling it as “positive” or “negative” [12]. Only one small study has identified a positive association between exposure on TikTok to content that promotes a non-judgmental attitude toward the body and prioritizes functionality over appearance in promoting a more positive body image and an improvement in mood [13].

Current evidence supports the need for targeted interventions focusing on media literacy education and content-specific approaches rather than simply limiting time spent on social media platforms. The type of content consumed, rather than the amount of time spent on social media, is most strongly associated with body image disturbances and disordered eating behavior [14]. The present study represents one component of the broader SWATCH (Social Withdrawal And TeCno-mediated mental Health issues) research project, which aims to investigate emerging web-mediated mental health phenomena associated with digital technologies and social media use. Within this framework, the current analysis specifically focuses on the relationship between SNS use, eating-related content consumption, and vulnerability to eating and feeding disorders. The present research aimed to examine patterns of SNS engagement among young people, with particular attention to the consumption of food-related content, body satisfaction, and body image concerns associated with SNS use. In addition, the study investigated the prevalence of FED-related vulnerability and disordered eating behaviors among individuals who follow pages, profiles, or communities focused on nutrition and eating habits on these platforms. The main objective of the study was to identify which SNS platforms are more frequently used by individuals vulnerable to FEDs and body image dissatisfaction, in order to inform the potential development of prevention and educational interventions delivered through social media. Furthermore, the study aimed to explore socio-demographic characteristics and SNS-related factors associated with these patterns of use, which may help guide the design of targeted prevention campaigns addressing food and nutrition issues among young people through SNS.

## 2. Materials and Methods

### 2.1. Study Design and Recruitment Strategies

A cross-sectional Italian population-based observational study was conducted by designing a questionnaire, which was distributed via an online survey using the EUSurvey platform (https://ec.europa.eu/eusurvey/home/welcome) by using a snowball sampling recruitment strategy. The survey was distributed on various social media platforms, with links to participate in the study also shared via WhatsApp, Telegram, and Instagram. On WhatsApp, the survey was distributed by word of mouth, through private groups and individual chats. Finally, on Instagram and Telegram, the survey was distributed with the help of a medical students’ page and through the personal profile of the researchers. The inclusion criteria for the study were as follows: (a) subjects aged between 18 and 35; (b) having provided consent to participate in the study and authorization to process their personal data for research purposes. No exclusion criteria were set. Participation was anonymous and voluntary without monetary or other incentives. All participants gave informed consent to take part in the study. The study was conducted in accordance with the ethical principles outlined in the Declaration of Helsinki and according to the guidelines for Good Clinical Practice (GCP) [15], following the approval by the local Institutional Review Board of the Department of Experimental and Clinical Medicine, Polytechnic University of Marche (protocol code ACPS-D-21-00347, 28 September 2021).

### 2.2. Survey Design

The survey development was completed on 26 November 2023, and questionnaires were distributed and collected during the period from 30 November 2023 to 30 April 2024. In accordance with the guidelines for personal data processing adopted by the Italian Data Protection Authority with resolution no. 52 of 24 July 2008 (Official Gazette no. 190 of 14 August 2008) and in conformity with Article 13 of the European General Data Protection Regulation EU 2016/679, collectively referred to as “Privacy Regulations”, the questionnaire is preceded by a module for data processing authorization in compliance with the Italian privacy law (Legislative Decree 196/03).

The questionnaire is constituted of four sections. The first section of the questionnaire collects key sociodemographic data and comprises 7 items (3 multiple-choice and 4 open-ended questions). The second section collects information regarding SNS usage and consists of 12 items composed of 6 dichotomous-response questions and the 6-item Bergen Social Media Addiction Scale (BSMAS) [16]. The third section aimed to investigate the presence of FEDs and/or body dysmorphic disorder, through the tool of SCOFF (5-item dichotomous questions) [17,18] as well as 5 items derived by the Body Uneasiness Test (BUT) [19]. The fourth section comprised more specific questions regarding the type of pages followed on SNS; specifically, regarding adequate dietary lifestyle, social profiles focused on fitness promotion, pages promoting unbalanced eating styles (i.e., prolonged fasting, binge eating, etc.), and profiles focused on body positivity and/or body neutrality. Finally, the questionnaire also comprises an optional section constituted by pop-up items which are accessible to participants only in case of a positive reply regarding the knowledge of Mukbang phenomenon, an SNS-mediated phenomenon consisting of a compulsive watching of a subject eating large quantities of food on a screen. This optional section is constituted by items derived by the Mukbang Addiction Scale (MAS) [20,21] (Supplementary File S1 and S2).

### 2.3. Measurements

The first section of the questionnaire (i.e., sociodemographic data form) investigated the following variables: age, sex, gender identity, height, weight, student status (yes/no), educational level, occupational status, marital status. The Body Mass Index (BMI) was calculated using height and weight.

The second section, dedicated to SNS use, included the Italian version of the Bergen Social Media Addiction Scale (BSMAS), consisting of six items, a tool for screening SNS addiction [16]. The instrument assesses problematic social media use (PSMU), based on the core components of addiction (i.e., salience, mood modification, tolerance, withdrawal, conflict, and relapse) [22]. The scale is rated on a 5-point Likert scale ranging from 1 “very rarely” to 5 “very often”. The scale refers to experiences related to social media use within a time frame of 12 months. The highest BSMAS total score is the highest propensity to develop problematic social media use. We adopted a cutoff of 19 to discriminate between problematic versus non-problematic use of social media (PSMU versus notPSMU), according to the study [23]. In our study, BSMAS displays a good internal reliability (Cronbach’s α = 0.788).

The third part contains the SCOFF, a FED screening questionnaire consisting of five dichotomous questions (yes/no), validated for routine use in patients at risk of FEDs [17,18]. The tool has excellent sensitivity of 100% in identifying individuals affected by anorexia nervosa and bulimia nervosa and good specificity of 87.5%. In the Italian validation, sensitivity is 97% and specificity is 87.3% [17,18]. The section also contains five items from the Body Uneasiness Test (BUT) [19], a test for assessing body image disorders, a self-report questionnaire consisting of 71 questions divided into two parts (BUT-A and BUT-B). BUT-A, from which the 5 items in the questionnaire were extracted, investigates weight phobia, concern about body image, avoidance, compulsive monitoring, detachment, and feelings of alienation from one’s own body; BUT-B examines concerns that the subject may have about specific parts of their body [19].

The optional section of the fourth section is constituted by the MAS which comprises 6 items on a 5-point Likert scale from “very rarely” to “very often” measuring six components of addiction (i.e., salience, conflict, withdrawal, mood modification, tolerance, and relapse) [20,21]. The study used the Italian validation of the MAS which found a good internal consistency (McDonald’s *ω*  =  0.89, Cronbach’s *α*  =  0.89) [21]. In our study, MAS also displays a good internal reliability (Cronbach’s α = 0.761).

### 2.4. Sample Size Calculation

The sample size was calculated a priori using the G*Power statistical software version 3.1. (Franz, Universitat Kiel, Kiel, Germany), based on internationally available prevalence data on FED samples from young people. Sensitivity analyses suggest a sample size of approximately 245 subjects in order to ensure a statistical power of 0.80 with 5% precision and a 95% confidence interval.

### 2.5. Statistical Analysis

Data analysis was conducted using the Statistical Package for Social Sciences (SPSS) for MacOS, Version 29.0 (2022, IBM Corporation, Chicago, IL, USA). Descriptive analyses were performed on the dataset, examining frequencies (*n*) and percentages (%) for categorical variables. The normality of continuous variable distribution was preliminarily assessed using the Kolmogorov–Smirnov normality test, skewness, and kurtosis measures. Normally distributed continuous variables were represented using mean and standard deviation (SD), while non-normally distributed variables were presented using median and confidence interval (CI). BMI was analyzed both as a continuous variable and categorized according to six BMI classes (underweight, normal weight, overweight, obesity classes I-II-III). The five BUT items were analyzed by transforming them into dummy variables (absent/present).

The total sample was categorized into two groups, based on SCOFF positivity: SCOFF+ (subjects with at least two out of five positive items) and SCOFF– (subjects with fewer than two out of five positive items). The two groups were compared based on socio-demographic characteristics (Section 1), general SNS usage variables (first part of Section 2), BSMAS total score (second part of Section 2), specific SNS usage variables for consulting nutrition and/or diet-related pages (Section 3), and MAS (Section 4). Specifically, independent sample *t*-tests were conducted to compare mean values and SD of continuous BSMAS and MAS variables between SCOFF+/SCOFF– groups. Additionally, Student’s *t*-tests or non-parametric Mann–Whitney U tests were performed to compare mean values and SD of continuous variables according to dichotomous categorical variables including socio-demographic factors, general SNS usage, specific usage for nutrition/diet-related page consultation, and for each of the five SCOFF items.

Categorical variables were compared using chi-square tests and Fisher’s exact tests. One-way analysis of variance (ANOVA) was conducted to compare mean values and SD of continuous variables between groups classified by gender identity and BMI categories. Where statistically significant differences were found, post hoc Bonferroni analyses and Tamhane’s test were performed. Pearson correlation analyses were conducted to examine potential associations between continuous variables investigated (SCOFF as quantitative variable with BSMAS, SCOFF as quantitative variable with MAS, and age, education level, and BMI as quantitative variables). A stepwise binary logistic regression analysis was run by selecting each SNS (TikTok, Instagram Facebook, Snapchat, BeReal) as dependent variable with all socio-demographic and FED-related variables, in order to identify risky and/or protective determinants on the preference of a specific SNS, with the goal to identify the preferred SNS for preventive and/or informative strategies. Furthermore, in order to identify potential risky and protective socio-demographic and SNS-related factors associated with the probability to develop FED, a stepwise binary logistic regression analysis (adjusted for sex) was run within all our sample. The estimated odds ratios (OR) along with the 95% of confidence intervals (95% CI), and standardized coefficient β values were generated for each variable.

## 3. Results

A total sample of 299 responses was finally collected; of these 16 were excluded due to refusal to participate in the research study for a final sample size of 283 (94.6% response rate).

### 3.1. Socio-Demographic and Clinical Characteristics of the Sample

The majority of the sample (69.3%; N = 196) consisted of women. The majority of the sample identified as cisgender (90.8%; N = 257), while a small proportion identified as transgender (3.9%; N = 11), non-binary (4.9%; N = 14), or agender (0.4%; N = 1). The median age of the sample was 23 (95% CI = 23.9–25.4), without significant sex-based differences (*p* = 0.113). The mean educational level was 14.3 years (SD = 2.9), without sex (*p* = 0.344), gender identity (*p* = 0.157) and BMI (*p* = 0.254) differences. Two-thirds of the sample constituted students (68.2%; N = 204). The mean BMI was 23.1 (SD = 3.7) Kg/m^2^, with sex-based differences (females display lower BMI compared to males, *p* < 0.001). Most of the sample (70.9%; N = 200) displayed a BMI in the normal range, while only 17 subjects (6%) were underweight (Table 1).

### 3.2. SNS-Related Characteristics of the Sample

Most of the participants declared using at least one SNS (98.6%; N = 279) without statistically significant differences based on sex (*p* = 0.639), age (*p* = 0.102), educational level (*p* = 0.100), or BMI (*p* = 0.094). Specifically, the majority of the sample reported using Instagram (93.6%; N = 265), followed by Facebook (46.3%; N = 131) and TikTok (45.9%; N = 130) (Table 2). Regarding the Instagram usage, there were no significant differences based on sex (*p* = 0.495), educational level (*p* = 0.918), and BMI (*p* = 0.673). However, younger participants displayed the greatest use of Instagram (*p* = 0.012). While TikTok seemed to be much more likely to be used by younger participants (*p* < 0.001), those with a lower educational level (*p* = 0.006), females [χ^2^(1) = 8.034; *p* = 0.006], and the student participants [χ^2^(1) = 6.102; *p* = 0.017]. No differences in TikTok use were found based on BMI (*p* = 0.243). Contrarily, participants who used Facebook were generally older (*p* < 0.001), with an average higher educational level (*p* < 0.001) and not students [χ^2^(1) = 10.915; *p* = 0.001]. No sex- (*p* = 0.519) and BMI-based (*p* = 0.321) differences were found.

In our sample, a mean BSMAS total score of 13.5 (SD = 4.5) was found, with a predominance of subjects without a PSMU (97.9%; N = 277) (Table 2). Female participants displayed significantly higher BSMAS scores compared to male counterparts [t(172.748) = −2.321; *p* = 0.021]. There were no significant differences based on BMI categories (*p* = 0.096). Participants who used at least one SNS displayed significantly higher BSMAS levels [t(3.625) = 5.982; *p* = 0.005]. Similarly, the participants who used Instagram or TikTok displayed significantly higher BSMAS levels [respectively, t(281) = 2.538; *p* = 0.012 and t(257.267) = 2.564; *p* = 0.011], but not other types of SNS. The participants positive to SCOFF displayed significantly higher BSMAS levels [t(277.232) = −3.719; *p* < 0.001], compared to those without FED positive screening.

### 3.3. FED-Related Characteristics of the Sample

**Figure 1:**
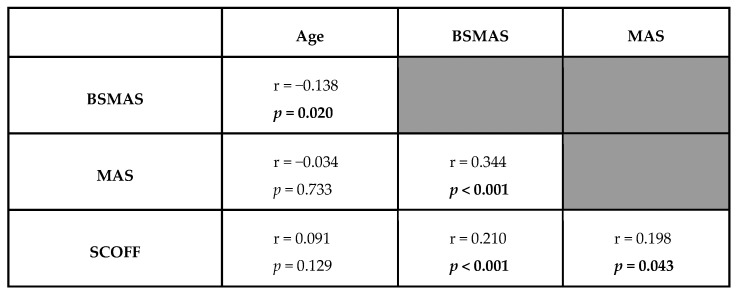
Pearson’s bivariate correlations across all quantitative variables. **In bold significant *p*-values**.

Most participants declared a negative FED history (86.6%; N = 245). Regarding the SCOFF screening questionnaire, most of the participants positively declared “I have felt disgusted because I was unpleasantly full” (62.2%; N = 176), while for the remaining four SCOFF questions, positivity did not exceed half of the sample. Overall, the sample was constituted by 55.8% (N = 158) of participants with a positive SCOFF screening (i.e., at least two positive questions at SCOFF) while 44.2% (N = 125) had a negative SCOFF screening. Two groups (SCOFF+ versus SCOFF–) were homogeneous in terms of age distribution (*p* = 0.266). Female participants were more represented among subjects with positive SCOFF [χ^2^(1) = 12.397; *p* < 0.001]. Both samples were homogeneous in terms of BMI category distribution (*p* = 0.113), gender identity (*p* = 0.686), SNS use (*p* = 0.596), except for the social TikTok, mainly used by participants positive to SCOFF [χ^2^(1) = 10.392; *p* = 0.002]. Figure 1 shows the correlations between SCOFF, BSMAS, and MAS.

Participants were also asked to reply to five questions selected by the BUT on body image. On these questions, no significant differences were found with respect to the variable “use of any SNS”. Among participants using Instagram, a greater positivity was found to the item “*My physical appearance is disappointing compared to my ideal image*” [χ^2^(1) = 5.559; *p* = 0.031]. While participants using TikTok mainly responded positively to the following items “*I spend a lot of time in front of the mirror*” [χ^2^(1) = 7.166; *p* = 0.011]; “*I am terrified of gaining weight*” [χ^2^(1) = 6.041; *p* = 0.019]; “*My physical appearance is disappointing compared to my ideal image*” [χ^2^(1) = 8.138; *p* = 0.005]. When comparing the sample divided by SCOFF, a predominance of positive responses among SCOFF-positive participants is observed in all five BUT items (*p* < 0.001). A stepwise logistic regression analysis was performed to ascertain all variables on the likelihood of using TikTok SNS. The model was statistically significant, χ^2^(1)  = 4.184, *p*  <  0.001. The model explained 6.8% (Nagelkerke R2) of the variance in TikTok use, by correctly classifying 59% of cases. According to the logistic regression model, TikTok use was significantly predicted by a positive SCOFF (OR = 1.9) and the positivity to the item “*I spend a lot of time in front of the mirror*” (OR = 1.9) (Table 3). However, the logistic regression analysis performed on the likelihood of using Instagram as SNS found a model statistically significant, χ^2^(1)  = 6.912, *p*  =  0.009, which explained 11.0% (Nagelkerke R2) of the variance in Instagram use by correctly classifying 93.6% of cases. According to the model, Instagram use was significantly predicted by the positivity to the item “*My physical appearance is disappointing compared to my ideal image*” (OR = 3.9) while following SNS profiles related to healthy eating habits and diet styles was protective by using Instagram (Table 3). No significant FED-related predictors were found for Facebook and Snapchat use. However, BeReal use was found to be significantly predicted only by the preference to look at/follow SNS profiles related to alternative eating styles (such as binge eating, prolonged fasting to reduce weight, and so on (Table 3).

### 3.4. SNS-Related Characteristics of the Sample Focussing on Eating Habits and/or Nutritional SNS-Delivered Topics

Most of our sample reported having watched and/or having followed SNS profiles related to the topic of a healthy eating style (68.9%; N = 195), with most respondents declaring that “*they encourage me, they help me take care of my health*” (64.6%; N = 126). Among the respondents, a significant prevalence was observed among female participants [χ^2^(1) = 9.282; *p* = 0.003], those who are currently studying [χ^2^(1) = 5.831; *p* = 0.022], users of Instagram [χ^2^(1) = 5.368; *p* = 0.032] but not of other SNS, participants positive to SCOFF [χ^2^(1) = 8.286; *p* = 0.005], those who declared “*I am terrified of gaining weight*” [χ^2^(1) = 13.601; *p* = 0.005]. *p* < 0.001], and that “*my physical appearance is disappointing compared to my ideal image*” [χ^2^(1) = 5.023; *p* = 0.026].

Most of our sample reported having watched and/or followed SNS profiles related to fitness (74.2%; n = 210), with most respondents declaring that “*they encourage me, help me take care of my health*” (68.1%; n = 143). Among the respondents, a significant prevalence of participants reported “*I sometimes felt fat even if others said I was too thin*” [χ^2^(1) = 9.136; *p* = 0.002], “*I spend a lot of time in front of the mirror*” [χ^2^(1) = 4.101; *p* = 0.048] and “*I am terrified of gaining weight*” [χ^2^(1) = 5.200; *p* = 0.026].

A small percentage of respondents reported watching and/or following SNS profiles promoting unbalanced eating habits (e.g., binge eating, prolonged fasting, etc.) (12%; n = 34). Almost half of our sample reported watching and/or following SNS profiles that promote specific movements such as body positivity and/or body neutrality movements (49.1%; n = 139). Among the latter participants, most of the respondents declared that “*they encourage me, they help me take care of my health*” (60.4%; n = 84). Among the respondents, there was a significant prevalence of female participants [χ^2^(1) = 51.068; *p* < 0.001] and those positive to SCOFF [χ^2^(1) = 10.288; *p* = 0.002], without a significant prevalence of an SNS, except for a trend among BeReal users (*p* = 0.063). A significant prevalence was also observed among participants who reported having been “*worried about having lost control over how much they had eaten*” [χ^2^(1) = 8.286, *p* = 0.005], who declared that they were “*terrified of gaining weight*” [χ^2^(1) = 10.686, *p* = 0.001], and who reported that “*food dominates my life*” [χ^2^(1) = 4.236, *p* = 0.045].

Only a third of the participants declared having watched a Mukbang on SNS (37.1%; N = 105); most of them reported once/year (45.7%; N = 48) or once/month (31.4%; N = 33), with an average of 8.7 (SD = 8.7) minutes of video. No significant differences in MAS total score was found among the two SCOFF groups (*p* = 0.599). Most of our sample declared to have never eaten while watching a Mukbang (64.8%; N = 68). Mukbang viewers are mainly non-TikTok users [χ^2^(1) = 23.822; *p* < 0.001] but SCOFF-positive subjects [χ^2^(1) = 12.694; *p* < 0.001]. However, it seems to be more used among those who declare “*to have felt disgusted because unpleasantly full*” [χ^2^(1) = 6.059; *p* = 0.016] and “*to have worried about having lost control over how much they had eaten*” [χ^2^(1) = 8.271; *p* = 0.005]. In our sample the mean MAS value was 7.1 (SD = 2.3), without any significant differences between the two SCOFF groups (*p* = 0.118) (Table 2).

A stepwise logistic regression analysis was performed to examine whether SNS-related variables were associated with the likelihood of screening positive on the SCOFF, initially run in the total sample, then run by using two separate analyses adjusting for confounding factors (i.e., sex) (Table 4). In the total sample, the logistic regression model was statistically significant, χ^2^(1)  = 4.049, *p*  =  0.044. The model explained 14.9% (Nagelkerke R2) of the variance in SCOFF positivity and correctly classified 66.1% of cases. According to the logistic regression model, FED risk was significantly predicted by TikTok use (OR = 1.7), higher BSMAS scores (OR = 1.1), SNS-related behaviors such as looking at SNS profiles related to body positivity and/or neutrality (OR = 1.9) and looking at Mukbang on SNS (OR = 1.8). In the female sample, the logistic regression model was statistically significant, χ^2^(1)  =  8.106, *p*  =  0.004. The model explained 14.9% (Nagelkerke R2) of the variance in SCOFF positivity and correctly classified 69.4% of cases. According to the logistic regression model, FED risk was significantly predicted by SNS-related behaviors such as looking at SNS profiles related to eating habits (OR = 2.6) and looking at Mukbang on SNS (OR = 3.2), while in the male sample, the logistic regression model was statistically significant, χ^2^(1)  =  4.262, *p*  =  0.039. The model explained only 6.5% (Nagelkerke R2) of the variance in SCOFF positivity and correctly classified 63.2% of cases. According to the logistic regression model, FED risk was significantly predicted only by higher BSMAS scores.

## 4. Discussion

To the best of our knowledge, our study represents one of the first designed to investigate the use of SNS as an educational and informative tool for the promotion of healthy eating and/or nutritional lifestyles and habits, through the recruitment of a non-clinical sample from the general population. The main goal of the study was to identify which SNS could be more effectively used in primary and secondary prevention strategies for FEDs in a non-clinical sample recruited from the general population, classified based on the SCOFF screening tool for FEDs, the type of SNS use, and both in general and specifically in the search/consumption of content related to eating and/or nutritional styles. Given the epidemiological distribution of FED onset typically in the young population, the study was mainly addressed to a sample of women aged between 18 and 35 years. The recruited sample effectively intercepted a population of both vulnerable subjects and non-vulnerable subjects, allowing a comparison between the two subgroups, across different socio-demographic variables and those correlated with the use of SNS (in general and specifically associated with eating and/or nutritional styles). The study investigated both the extent of SNS use (more specifically in association with the eating dimension and/or satisfaction with one’s body image) and the dimension of the FED phenomenon and altered eating/nutritional styles in a sample of young people who consult pages, profiles, etc., on SNS dedicated to the topics ‘*eating*’, ‘*eating habits*’, and ‘*eating behaviors*’. The final goal was to identify which SNS could be more attractive to the population of young people positive on the SCOFF and for the subgroup of participants who display a polarized ideation regarding their body image and/or dissatisfaction with their body shape and/or who prefer the search for content on SNS regarding “unhealthy/unbalanced” eating and/or nutritional lifestyles.

Most of the sample declared using at least one SNS, mainly Instagram, followed by Facebook and TikTok. The youngest participants reported preferentially using TikTok and Instagram, while female participants seem to primarily prefer TikTok. These results are in line with previous studies on SNS preferences of youngsters, supporting the adequacy of our sampling strategy [24]. Those participants using TikTok and Instagram displayed significantly higher scores at the BSMAS, which is indicative of a higher risk for PSMU. This could be explained by the immersive functionality offered by this SNS, in particular TikTok. The more time people spend online, the more the system collects psychological data to personalize content via AI, encouraging them to stay connected [25]. This mechanism satisfies their need for gratification, attention, and self-affirmation, helping to create addictive dynamics, especially among teenagers and young adults, who are the most vulnerable to negative effects on their mental balance [25]. Participants positive to SCOFF screening are much more likely to use TikTok and Instagram, among all the SNS. In particular, according to our findings, TikTok seems to be preferred by those subjects most vulnerable to FEDs, particularly the female and younger population in our sample, as well as by subjects who report perceiving their physical appearance as disappointing compared to their body image, those who express feelings of terror regarding the possibility of gaining weight, and those who report spending a lot of time in front of the mirror. Therefore, TikTok seems to be preferred by both subjects vulnerable to FEDs (SCOFF-positive) and by those who manifest distortions and/or dissatisfaction with their body image and/or shape. These findings also seem to be confirmed by the literature published so far, which has identified an association between exposure to idealized and perceived as attractive bodies on TikTok, the emergence of alterations in eating and/or nutritional style, and the appearance of FED-related symptomatology [26]. Furthermore, some studies confirm that TikTok is preferentially used for the consumption of promotional videos on diet-related products, including products for weight loss, for increasing muscle mass, and detox products, while in most cases not based on scientifically correct information [27]. This result can be explained by the fact that TikTok can be particularly appealing to pro-eating disorder communities because its personalized algorithm tends to show more and more content about dieting, body image, and eating disorders, creating a “bubble” of similar content [28,29,30,31]. In addition, the short, visual video format makes messages such as body checks or “what I eat in a day” much more immediate and easier to imitate [28,29,30,31]. Finally, the platform facilitates the creation of very active communities, where users find mutual support and normalization of the disorder, reinforcing their sense of belonging [28,29,30,31]. Instagram, a photo-based SNS, on the other hand, seems to be preferred only by subjects who report perceiving their physical appearance as disappointing compared to their ideal image. Although Instagram use did not significantly predict SCOFF positivity, it was significantly associated with body image dissatisfaction, suggesting that this platform may be particularly relevant for body image related vulnerability. Evidence shows that Instagram use is associated with poorer body image and self-esteem, largely mediated by appearance-based social comparison processes [32]. In particular, browsing Instagram predicts lower body appreciation through upward comparison with influencers and idealized bodies [33]. Experimental and meta-analytic evidence further indicates that exposure to idealized social media imagery increases body dissatisfaction via appearance comparison mechanisms [32,33,34,35].

Therefore, from our findings, it seems that TikTok can plausibly intercept both subjects at risk for FED and/or body dysmorphic disorder, becoming a potential tool to use in mediating informative and/or educational content for preventive purposes aimed at this population of individuals. However, our findings do not directly demonstrate that these platforms promote healthy eating habits; rather, they suggest that SNS such as TikTok and Instagram may represent strategic channels through which preventive and educational content could be delivered to vulnerable populations. Previous studies already suggested that both the SNS TikTok and Instagram could indeed be more frequently used by subjects with FED and/or alterations of their body image [36,37]. In particular, previous studies suggested that the use of the SNS TikTok can help promote remission from FEDs in a group of female adolescents [38,39]. Furthermore, according to our study, about two-thirds of the sample reported having watched and/or followed SNS profiles concerning the topic of “*correct eating habits*”, recognizing that, in most cases, they help motivate them to take care of their health and body. Also, regarding this type of SNS use, in our sample, the majority of respondents were female. Students seem to be the main consumers of such content on SNS, as well as SCOFF-positive subjects, those who report expressing feelings/fears related to the fear of gaining weight or report that their physical appearance is disappointing compared to their ideal image. Hence, these findings suggest, also in this case, the possibility of implementing prevention strategies mediated by SNS through accounts and profiles/pages whose main topic is eating and/or nutritional styles. According to our results, this topic seems to be most sought after by individuals who report mainly using the SNS Instagram, suggesting a second SNS channel to use in defining preventive strategies for FEDs. From a practical perspective, these findings suggest several potential applications for education and prevention strategies delivered through SNS. For example, public health institutions, clinicians, and researchers could use platforms such as TikTok and Instagram to disseminate short educational videos promoting balanced eating habits, media literacy regarding idealized body images, and early warning signs of eating disorders. In addition, collaborations with health professionals, dietitians, and credible influencers could help increase the visibility of evidence-based nutritional information and counteract misleading diet-related content. Furthermore, targeted campaigns addressing vulnerable populations could focus on promoting body acceptance, critical evaluation of online content, and healthy relationships with food. Such approaches may represent a promising way to reach young individuals through the same digital environments in which eating-related content is commonly consumed. However, it is important to ensure that this habit does not cause excessive dietary rigidity and lead to cases of orthorexia nervosa [40].

Furthermore, according to our study, participants who use SNS for the consultation/consumption of content mainly related to fitness are instead more represented by subjects who report expressing feelings related to the fear of gaining weight, those who spend a lot of time in front of the mirror, and those who exhibit ideation related to body dysmorphia. These results are in line with already published literature [9,41,42,43,44]. Conversely, there is no significant predominance in the FED-vulnerable sample compared to the non-vulnerable sample. In this case, it was not possible to highlight a predominant SNS. Indeed, our sample does not appear to be very representative of the portion of individuals who use SNS in the search for unbalanced eating styles (e.g., binge eating, prolonged fasting, etc.), confirming that the sample we intercepted probably does not belong to the clinical sample. Interestingly, almost half of our sample reports participants using SNS to follow/consume contents that promote body positivity and/or neutrality, stating as the main reason that they have an encouraging and/or motivating effect in taking care of their health. Also in this case, the majority of respondents were female and vulnerable to FEDs (SCOFF-positive). Although a predominant SNS was not identified, our data confirms a trend (albeit non-significant) among users of BeReal. Furthermore, the main consumers of body positivity and/or neutrality content on SNS seem to be those who report feelings related to the fear of gaining weight, concerns regarding the fear of losing control over eating, and with polarized ideation on eating, suggesting that such content can effectively intercept subjects at risk of developing FED and/or body dysmorphia [14,45].

Finally, on exploring the Mukbang phenomenon which is mainly consumed through the use of SNSs that offer the possibility of viewing videos, in our sample this phenomenon seems to be most consumed by SCOFF-positive participants. Furthermore, Mukbang consumers appear to be more represented by those who prefer not to eat while watching such binges on SNS, those who express feelings of disgust related to the sensation of feeling unpleasantly full from the food ingested, and those who express fears related to the loss of control over eating, suggesting that this SNS-mediated behavior may likely be sought after by those who exhibit emotional dysregulation and/or impulse dyscontrol in eating and/or bulimic and/or purging, or potentially restrictive behaviors. The association between Mukbang and FED has also been investigated and confirmed in other studies [20,46]. In this case, it was not possible to identify a preferential SNS, although subjects who report using TikTok preferentially are those who least frequently report using SNS to watch Mukbang, suggesting that preventive programs should probably be differentiated based on the different targets of the FED spectrum conditions.

Despite the above-mentioned promising findings and the clinically helpful insights provided regarding the type of SNS relevant to be addressed to build education with informative and preventive strategies to promote healthy nutritional and eating lifestyles through the use of SNS platforms mostly consulted by FED-sensitive young individuals, a set of limitations should be discussed to draw definitive generalizable findings. First, our sample is mainly represented by female subjects (who represent two-thirds of the total sample). Hence, it would be possible to hypothesize that the results emerging from our study may not be generalizable to a male sample (therefore, for example, subjects with orthorexia and/or reverse anorexia nervosa may not have been intercepted). Second, our sample is not very representative of the LBTQI+ population, which could be much more likely associated with FEDs [47,48,49]. Third, despite our sample of mainly recruited young adult subjects (18–24 years) being mainly FED epidemiologically represented among adolescents (aged less than 18 years-old), our data could not be easily generalized to the sample of adolescents and/or pre-adolescents. Therefore, a further study should also explore differences in SNS use between pre-adolescents and/or adolescents compared to our findings which refer mainly to young adults. Fourth, the cross-sectional nature of the study design does not allow for collecting longitudinal data that can evaluate the effectiveness in terms of prevention of the eventual provision of educational and/or informative interventions on eating and/or nutrition to this vulnerable population through the use of a specific SNS. This is in order to confirm that the selection of an SNS (TikTok and/or Instagram) actually represents the most indicated and effective tool in the provision of content for the promotion of health and eating and nutritional well-being. Furthermore, our data did not collect specific information regarding the possible methods of providing such promotional/preventive content to the vulnerable population (e.g., the modality of video pills on SNS, their duration, which types of SNS are more indicated based on vulnerability to a specific FED spectrum, etc.).

Based on these preliminary data, further longitudinal studies could be suggested, involving both a sample of subjects in the pre-adolescent and adolescent age group and a sample homogeneously distributed by sex but also representative of the LBTQI+ community. This can identify which specific individual and psychological characteristics might suggest greater attractiveness and effectiveness in the use of prevention programs mediated by SNS. Our sample included a very small proportion of participants identifying as transgender, non-binary, or other gender identities. Therefore, it was not possible to conduct meaningful analyses exploring the relationship between gender identity and FED-related variables. At the same time, future studies should be conducted on populations from different countries to confirm our findings, as well as selecting specific populations that may be particularly at risk, such as university students [50,51,52,53]. Furthermore, it appears useful to investigate the reasons linked to the greater use of the SNS TikTok by subjects most vulnerable to FEDs (SCOFF-positive) and of the SNS Instagram by those who show a greater vulnerability to body dysmorphic disorder. This is in order to understand if there are preferential SNS channels to take into account in differentiating primary and secondary prevention campaigns for ED. Finally, given the prevalence of the Mukbang phenomenon among SCOFF-positive participants, this SNS-mediated entity should be adequately studied and understood within the FED spectrum, as it could be hypothesized that it may function as a sort of coping strategy regarding the impulse to eat in subjects vulnerable to developing an FED. The field of investigation of new psychopathological and psychological trends mediated by SNS, in fact, represents a new interesting research area in the psychological and psychiatric field. However, it can actually be hypothesized that new technologies and new web-mediated tools, such as SNS, can represent not only therapeutic tools but also be employed with the aim of implementing educational and informative prevention strategies for the development of FED, from a nutritional and alimentary point of view.

## 5. Conclusions

Our study was able to recruit a non-clinical population, vulnerable vs. non-vulnerable to FED and/or unbalanced eating and/or nutritional styles. The study investigated the type of use of SNS, both in general and specifically in the search/consumption of content related to particular eating and/or nutritional styles, in order to identify the best strategies for implementing primary and secondary prevention programs across various SNS. Our data allowed us to identify the SNS TikTok as a possible communication channel to be used in the delivery and mediation of informative and/or educational content for preventive purposes, as it seems capable of intercepting both subjects at risk of FEDs and those with body dysmorphic disorder. However, it also emerged that prevention programs would be more effective if differentiated based on the specific FED, as there seems to be a preference for a certain SNS depending on the type of vulnerability. It would be useful to develop further studies to confirm our data and to verify if they can also be translated to a more homogeneous sample by sex and gender orientation, as well as a sample of pre-adolescents and adolescents and, in addition, to test and investigate the effectiveness of primary and secondary prevention strategies through the use of specific SNS communication channels.

## Figures and Tables

**Table 1:** Socio-demographic and clinical characteristics of the sample stratified according to SCOFF.

	Total Sample (*n* = 283)	SCOFF+ (*n* = 158)	SCOFF– (*n* = 125)	*p*-Value
**Sex**				
Males, N (%)	87 (30.7%)	35 (22.2%)	52 (41.6%)	χ^2^ = 12.397 ***p* < 0.001**
Females, N (%)	196 (69.3%)	123 (77.8%)	73 (58.4%)	
**Gender Identity**				
Cisgender, N (%)	257 (90.8%)	145 (91.8%)	112 (89.6%)	χ^2^ = 1.790 *p* = 0.686
Transgender, N (%)	11 (3.9%)	6 (3.8%)	5 (4.0%)	
Agender, N (%)	1 (0.4%)	1 (0.6%)	0 (0.0%)	
Non-binary, N (%)	14 (4.9%)	6 (3.8%)	8 (6.4%)	
**Age, years**				
Age, M (SD)	24.5 (5.9)	24.9 (6.4)	24.1 (5.1)	*p* = 0.956 *
**Educational level, years**				
Education, M (SD)	14.3 (2.9)	14.5 (3.1)	14.1 (2.7)	*p* = 0.232 *
**Occupational status**				
Student, yes, N (%)	204 (72.1%)	116 (56.9%)	88 (43.1%)	χ^2^ = 0.316 *p* = 0.334
**Anthropometric data**				
Underweight, N (%)	17 (6.0%)	9 (5.7%)	8 (6.4%)	χ^2^ = 7.910 *p* = 0.095
Normal weight, N (%)	200 (70.9%)	103 (65.2%)	97 (77.6%)	
Overweight, N (%)	51 (17.7%)	35 (22.2%)	16 (12.8%)	
Obesity I level, N (%)	12 (4.3%)	8 (5.1%)	4 (3.2%)	
Obesity II level, N (%)	3 (1.1%)	3 (1.9%)	0 (0%)	
BMI, M (SD)	23.1 (3.7)	23.6 (4.1)	22.5 (3.1)	*p* = 0.107 *
**FED history**				
FED current, yes, N (%)	9 (3.2%)	9 (5.7%)	0 (0%)	χ^2^ = 17.138 ***p* < 0.001**
FED past, yes, N (%)	29 (10.2%)	25 (15.8%)	4 (3.2%)	

N = sample size, M = mean; SD = standard deviation; SCOFF = Sick, Control, One stone, Fat, Food; FED = Feeding and Eating Disorders; BMI = Body Mass Index. * Mann–Whitney’s U test on independent samples. **In bold significant *p*-values.**

**Table 2:** SNS-related characteristics of the sample stratified according to SCOFF.

	Total Sample (*n* = 283)	SCOFF+ (*n* = 158)	SCOFF– (*n* = 125)	*p*-Value
SNS use, yes, N (%)	279 (98.6%)	153 (98.7%)	123 (98.4%)	χ^2^ = 0.056 *p* = 0.596
Instagram use, yes, N (%)	265 (93.6%)	147 (93%)	118 (94.4%)	χ^2^ = 0.217 *p* = 0.807
Tik Tok use, yes, N (%)	153 (54.1%)	86 (54.4%)	44 (35.2%)	χ^2^ = 10.392 ***p* = 0.002**
BeReal use, yes, N (%)	62 (21.9%)	39 (24.7%)	23 (18.4%)	χ^2^ = 1.611 *p* = 0.247
Facebook use, yes, N (%)	131 (46.3%)	69 (43.7%)	62 (49.6%)	χ^2^ = 0.987 *p* = 0.339
Snapchat use, yes, N (%)	12 (4.2%)	7 (4.4%)	5 (4.0%)	χ^2^ = 0.032 *p* = 0.551
**SNS-related scales**				
BSMAS, M (SD)	13.5 (4.5)	14.4 (4.6)	12.5 (4.1)	*t* = −3.719 ***p* < 0.001**
PSMU, yes, N (%)	6 (2.1%)	4 (2.5%)	2 (1.6%)	χ^2^ = 0.292 *p* = 0.697
**SNS behaviors related to eating and/or nutritional contents**				
Looking at SNS profiles related to the topic of healthy eating and/or dietary style, yes, N (%)	195 (68.9%)	120 (75.9%)	75 (60%)	χ^2^ = 8.286 ***p* = 0.005**
Looking at SNS profiles related to the topic of fitness, yes, N (%)	210 (74.2%)	125 (79.1%)	85 (68%)	χ^2^ = 4.503 ***p* = 0.040**
Looking at SNS profiles related to the promotion of alternative eating style, yes, N (%)	34 (12.0%)	24 (15.2%)	10 (8.0%)	χ^2^ = 3.413 *p* = 0.069
Looking at SNS profiles promoting positive/neutrality movement, yes, N (%)	139 (49.1%)	91 (57.6%)	48 (38.4%)	χ^2^ = 10.288 ***p* = 0.002**
**SNS behaviors related to Mukbang phenomenon**				
Looking at SNS profiles related to the topic of Mukbang, yes, N (%)	105 (37.1%)	73 (46.2%)	32 (25.6%)	χ^2^ = 12.694 ***p* < 0.001**
MAS, M (SD)	7.1 (2.3)	7.3 (2.6)	6.6 (1.1)	*t* = −1.577 *p* = 0.118

N = sample size, M = mean; SD = standard deviation; BSMAS: Bergen Social Media Addiction Scale; MAS: Mukbang Addiction Scale; PSMU: Problematic Social Media Use; SCOFF = Sick, Control, One stone, Fat, Food. **In bold significant *p*-values.**

**Table 3:** Stepwise binary logistic regression analysis predicting the use of a specific SNS based on FED-related variables.

Tiktok Use	B	SE	Wald	df	*p*-Value	Exp (B)	95% CI Exp (B)	
SCOFF positive	0.685	0.252	7.392	1	**0.007**	1.984	1.211	3.251
I spend a lot of time in front of the mirror, yes	0.674	0.333	4.086	1	**0.043**	1.962	1.021	3.772
**Instagram use**	**B**	**SE**	**Wald**	**df**	***p*** **-value**	**Exp (B)**	**95% CI Exp (B)**	
My physical appearance is disappointing compared to my ideal image, yes	1.367	0.515	7.033	1	**0.008**	3.923	1.429	10.775
Looking at SNS profiles related to the topic of healthy eating and/or dietary style, yes	−1.351	0.515	6.870	1	**0.009**	0.259	0.094	0.711
**BeReal use**	**B**	**SE**	**Wald**	**df**	***p*** **-value**	**Exp (B)**	**95% CI Exp (B)**	
Feeling to be fat even if others told you that you were too thin, yes	−0.882	0.299	8.734	1	**0.003**	0.414	0.231	0.743
Looking at SNS profiles related to the promotion of alternative eating style, yes	1.307	0.631	4.296	1	**0.038**	3.697	1.074	12.726

SE: Standard Error; CI: Confidence Interval; SCOFF = Sick, Control, One stone, Fat, Food. **In bold significant *p*-values.**

**Table 4:** Stepwise binary logistic regression analysis predicting the risk of developing an FED (as dependent variable, according to the SCOFF)—total sample.

Total Sample	B	SE	Wald	df	*p*-Value	Exp (B)	95% CI Exp (B)	
TikTok use, yes	0.532	0.265	4.040	1	**0.044**	1.702	1.013	2.860
BSMAS total score	0.078	0.031	6.457	1	**0.011**	1.082	1.018	1.149
Looking at SNS profiles related to the topic of body positivity and/or neutrality style, yes	0.670	0.255	6.908	1	**0.009**	1.955	1.186	3.222
Looking at SNS profiles related to the topic of Mukbang, yes	0.598	0.280	4.557	1	**0.033**	1.818	1.050	3.148
**Only female sample**	**B**	**SE**	**Wald**	**df**	***p*** **-value**	**Exp (B)**	**95% CI Exp (B)**	
Looking at SNS profiles related to the topic of eating style, yes	0.985	0.348	7.997	1	**0.005**	2.679	1.353	5.304
Looking at SNS profiles related to the topic of Mukbang, yes	1.174	0.342	11.780	1	**<0.001**	3.235	1.655	6.324
**Only male sample**	**B**	**SE**	**Wald**	**df**	***p*** **-value**	**Exp (B)**	**95% CI Exp (B)**	
BSMAS	0.109	0.055	3.926	1	**0.048**	1.115	1.001	1.241

SE: Standard Error; CI: Confidence Interval; SNS: Social Network Sites; BSMAS: Bergen Social Media Addiction Scale; SCOFF = Sick, Control, One stone, Fat, Food. **In bold significant *p*-values.**

## Data Availability

Data are available on request due to restrictions (privacy reasons).

## Supplementary Materials

The following supporting information can be downloaded at: https://www.mdpi.com/article/10.3390/brainsci16040379/s1, Supplementary File S1. Survey questionnaire (Italian version); Supplementary File S2. Survey questionnaire (English version).
