# Supplementary material for: TikTok and Instagram as Putative Social Media in Promoting Healthy Eating Habits in Youths At-Risk for Eating/Feeding Disorders and Body Image Dissatisfaction
Source: Brain Sci. 2026 Mar 30;16(4):379. doi: 10.3390/brainsci16040379 (PMC13113989; doi:10.3390/brainsci16040379)
Supplement: Supplementary file 1 [file brainsci-16-00379-s001.zip › brainsci-4186307-supplementary.pdf]

Supplementary File S1. Survey questionnaire (Italian version)

1. *Indichi la sua età (rispondere utilizzando solo numeri, es. 18)*
2. *Sesso alla nascita*
   - *Maschio*
   - *Femmina*
3. *Identità di genere*
   - *Cisgender*
   - *Transgendere*
   - *Agender*
   - *Non binario*
4. *Indichi la sua altezza in centimetri (esempio: se è alto/a 1 metro e 75 allora saranno 175 centimetri)*
5. *Indichi il suo peso espresso in chilogrammi (esempio: 70 kg)*
6. *Frequenta attualmente un corso di studi*
   - *Si*
   - *No*
7. *Indichi gli anni di scolarità compiuti (esempio: se attualmente sta frequentando il liceo allora saranno 8, se sta frequentando l'università 13, ecc.)*
8. *Utilizza qualche social network? (Esempio: TikTok, Instagram, ecc.)*
   - *Si*
   - *No*
9. *Quali social network utilizza maggiormente? Puoi segnare più di una risposta (si/no)*
   - *Instagram*
   - *TikTok*
   - *BeReal*
   - *Facebook*
   - *Snapchat*
10. *Di seguito sono riportate alcune domande sul rapporto con i social media e sull'uso che ne fa (Facebook, TikTok, ecc.). Per ciascuna domanda scelga la risposta che la descrive meglio. Durante l'ultimo anno con quale frequenza… (molto raramente, raramente, qualche volta, spesso, molto spesso)*
    - *ha trascorso molto tempo pensando hai social media o ha programmato di usarli?*
    - *Ha sentito il bisogno di usare sempre di più i social media?*
    - *Ha usato i social media per dimenticare i suoi problemi personali?*
    - *Ha provato a smettere di usare i social media senza riuscirci?*
    - *È diventato ansioso o agitato se le è stato proibito l'uso dei social media?*
    - *Ha utilizzato i social media così tanto che il loro uso ha avuto un impatto negativo sul suo lavoro/i suoi studi?*
11. *Le chiediamo di fornire un'unica risposta alle domande elencate qui di seguito (si/no)*
    - *Si è mai sentito/a disgustato/a perché sgradevolmente pieno/a*
    - *Si è mai preoccupato/a di aver perso il controllo*
    - *su quanto aveva mangiato*
    - *Ha perso recentemente più di 6 kg in un periodo di tre mesi*
    - *Le è mai capitato di sentirsi grasso/a anche se gli*
    - *altri le dicevano che era troppo magro/a*
    - *Affermerebbe che il cibo domina la sua via*

12. *Di seguito sono riportate alcune domande sul suo rapporto con la sua immagine corporea. Per ciascuna domanda scelga la risposta che la descrive meglio (mai, raramente, qualche volta, spesso, molto, sempre)*
    - *Trascorro molto tempo davanti allo specchio*
    - *Mi piaccio e gli abiti che nascondono le forme del mio corpo*
    - *Quando mi spoglio evito di guardarmi*
    - *Ho il terrore di ingrassare*
    - *Il mio aspetto fisico è deludente*
    - *rispetto alla mia immagine ideale*
13. *Le è mai capitato di guardare/seguire profili social inerenti al tema di un adeguato stile alimentare? (Si/no)*
    - *Se sì, come la fanno sentire: mi incoraggiano/ aiutano a prendermi cura della mia salute/ non mi suscitano nulla/ mi mettono a disagio, mi demoralizzano/ altro*
14. *Le è mai capitato di guardare/seguire profili social inerenti al tema fitness? (Si/no)*
    - *Se sì, come la fanno sentire: mi incoraggiano/ aiutano a prendermi cura della mia salute/ non mi suscitano nulla/ mi mettono a disagio, mi demoralizzano/ altro*
15. *Le è mai capitato di guardare/seguire profili social che promuovono stili alimentari alternativi? (es. abbuffate, digiuni prolungati per dimagrire, etc.) (Si/no)*
    - *Se sì, specificare quali*
    - *Se sì, come la fanno sentire: mi incoraggiano/ aiutano a prendermi cura della mia salute/ non mi suscitano nulla/ mi mettono a disagio, mi demoralizzano/ altro*
16. *Le è mai capitato di guardare/seguire profili social che promuovono movimenti come il body positivity e/o neutrality? (Si/no)*
    - *Se sì, come li ha conosciuti: amici, parenti, sui social, altro*
    - *Se sì, come la fanno sentire: mi incoraggiano/ aiutano a prendermi cura della mia salute/ non mi suscitano nulla/ mi mettono a disagio, mi demoralizzano/ altro*
17. *Hai mai visto sui social network un Mukbang? (Un video/diretta online in cui una persona mangia cibo) (si/no)*
    - *Se sì, quanto spesso guardi Mukbang? (Una volt all'anno, una volta al mese, una volta alla settimana, più volte alla settimana, ogni giorno)*
    - *Se sì, in media, per quanti minuti guardi video Mukbang?*
    - *Se sì, quanto spesso ti capita di magiare mentre stai guardando Mukbang? (Mai, quasi mai, qualche volta, spesso, sempre)*
    - *Se sì, durante l'ultimo anno con quale frequenza… (molto raramente, raramente, qualche vota, spesso, molto spesso)*
      - quante volte vi è capitato di pensare o programmare di guadare un Mukbang
      - quante volte avete sentito il bisogno di guardare il Mukbang sempre di più
      - quante volte ha guardato il Mukbang per dimenticare i problemi personali
      - quante volte avete cercato di ridurre la visione di Mukbang senza successo
      - quante volte siete diventati inquieti o preoccupati se vi è stato proibito di guardare Mukbang
      Quante volte vi è capitato di guardare il Mukbang così tanto da aver un impatto negativo sul suo lavoro/studio
18. *Soffre attualmente/ha sofferto in passato di un disturbo del comportamento alimentare? (Si/si, in passato/no)*
    - *Se sì/si, in passato, specificare il tipo di disturbo*

Supplementary File S2. Survey questionnaire (English version)

1. Please indicate your age (answer using only numbers, e.g., 18)

2. Please indicate your sex:

- *Male*
- *Female*

3. Please indicate your gender identity

- *Cisgender*
- *Transgender*
- *Agender*
- *Non-binary*

4. Please indicate your height in centimeters (example: if you are 1 meter and 75, then it is 175 centimeters)

5. Please indicate your weight expressed in kilograms (example: 70 kg)

6. Are you currently studying?

- *Yes*
- *No*

7. Please indicate the years of completed schooling (example: if you are currently attending high school, it will be 8, if you are attending university, 13, etc.)

8. Do you use any social networks? (Example: TikTok, Instagram, etc.)

- *Yes*
- *No*

9. Which social networks do you use most often? You can select more than one answer (yes/no)

- *Instagram*
- *TikTok*
- *BeReal*
- *Facebook*
- *Snapchat*

10. Below are some questions about your relationship with social media and your use of it (Facebook, TikTok, etc.). For each question, choose the answer that best describes you. During the last year, how often... (very rarely, rarely, sometimes, often, very often)

- *Have you spent a lot of time thinking about social media or planning to use it?*
- *Have you felt the need to use social media more and more?*
- *Have you used social media to forget your personal problems?*
- *Have you tried to stop using social media without success?*

- *Have you become anxious or agitated if you were prohibited from using social media?*
- *Have you used social media so much that its use has negatively impacted your work/studies?*

11. We ask you to provide a single answer to the questions listed below (yes/no)

- *Have you ever felt disgusted because you were unpleasantly full?*
- *Have you ever worried about losing control over how much you ate?*
- *Have you recently lost more than 6 kg in a three-month period?*
- *Have you ever felt fat even if others told you that you were too thin?*
- *Would you state that food dominates your life?*

12. Below are some questions about your relationship with your body image. For each question, choose the answer that best describes you (never, rarely, sometimes, often, very often, always)

- *I spend a lot of time in front of the mirror*
- *I like myself and the clothes that hide the shape of my body*
- *When I undress, I avoid looking at myself*
- *I have a fear of gaining weight*
- *My physical appearance is disappointing compared to my ideal image*

13. Have you ever looked at/followed social profiles related to the topic of an adequate eating style? (Yes/no)

- *If yes, how do they make you feel: they encourage/help me take care of my health/ they don't elicit anything/ they make me uncomfortable, they demoralize me/ other*

14. Have you ever looked at/followed social profiles related to the topic of fitness? (Yes/no)

- *If yes, how do they make you feel: they encourage/help me take care of my health/ they don't elicit anything/ they make me uncomfortable, they demoralize me/ other*

15. Have you ever looked at/followed social profiles that promote alternative eating styles? (e.g., binges, prolonged fasting to lose weight, etc.) (Yes/no)

- *If yes, specify which ones*
- *If yes, how do they make you feel: they encourage/help me take care of my health/ they don't elicit anything/ they make me uncomfortable, they demoralize me/ other*

16. Have you ever looked at/followed social profiles that promote movements like body positivity and/or neutrality? (Yes/no)

- *If yes, how did you learn about them: friends, relatives, on social media, other*
- *If yes, how do they make you feel: they encourage/help me take care of my health/ they don't elicit anything/ they make me uncomfortable, they demoralize me/ other*

17. Have you ever seen a Mukbang on social networks? (An online video/live stream in which a person eats food) (yes/no)

- *If yes, how often do you watch Mukbang? (Once a year, once a month, once a week, several times a week, every day)*

- *If yes, on average, for how many minutes do you watch Mukbang videos?*
- *If yes, how often do you eat while watching Mukbang? (Never, almost never, sometimes, often, always)*
- *If yes, during the last year how often... (very rarely, rarely, sometimes, often, very often)*
    - *how many times have you thought about or planned to watch a Mukbang*
    - *how many times have you felt the need to watch Mukbang more and more*
    - *how many times have you watched Mukbang to forget personal problems*
    - *how many times have you tried to reduce watching Mukbang without success*
    - *how many times have you become restless or worried if you were prohibited from watching Mukbang*
    - *How many times have you watched Mukbang so much that it had a negative impact on your work/study*

18. Do you currently suffer/have you suffered in the past from an eating disorder? (Yes/yes, in the past/no)

- *If yes/yes, in the past, specify the type of disorder*
